# Laser coagulation of blood vessels at 454 nm wavelength for neurosurgical interventions

**DOI:** 10.1117/1.JBO.30.7.078001

**Published:** 2025-07-16

**Authors:** Christina Giesen, Elisa Jarry, Lazar Bochvarov, Achim Lenenbach

**Affiliations:** Fraunhofer Institute for Laser Technology, Aachen, Germany

**Keywords:** photocoagulation, hemostasis, occlusion rate, blood vessel sealing, neurosurgery, optical coherence tomography

## Abstract

**Significance:**

In neurosurgery, where operations take place near tissue structures with high functionality, precise devices for microsurgical procedures such as blood vessel coagulation are crucial. Currently, bipolar forceps that deliver up to 60 W with high alternating current are used for vascular coagulation (hemostasis) to thermally seal blood vessels and stop bleeding. However, the high current can disturb electrophysiological monitoring and cause nerve damage from heat spread.

**Aim:**

Therefore, a safer and more efficient microsurgical procedure is required to seal individual blood vessels.

**Approach:**

Our approach uses a wavelength of 454 nm, which closely matches the hemoglobin absorption peak to directly heat the blood and avoid thermal damage to surrounding tissue. In experiments on blood vessels at the vascular tree of pig hearts, occlusion rates of different vessel diameters, the thermal damage, and the dynamics of the coagulation process using optical coherence tomography were investigated.

**Results:**

Our findings show that laser radiation of 454 nm wavelength can reliably coagulate vessels up to 400  μm in diameter with small thermal damage zones. Further research will be necessary to occlude larger vessels with a blood pressure of more than 120 mmHg.

**Conclusions:**

Overall, we present a laser process that can fundamentally improve the safety and operation time in neurosurgical interventions.

## 1 Introduction

Hemostasis during microsurgery is a critical life-sustaining aspect of surgical procedures and requires precise equipment and reliable processes. Particularly, in neurosurgery, where operations are performed in the vicinity of high-risk structures, such as cranial nerves and neurophysiological monitoring is used for assistance, the precision and low invasiveness of the surgical devices are crucial. Currently, surgeons operate with bipolar forceps that deliver up to 60 W using alternating currents at high frequencies in the radio frequency (RF) range (typically 100 kHz to 5 MHz) for hemostasis. The RF current induces oscillations of intracellular molecules, which result in tissue heating. Above 40°C to 60°C, proteins start to coagulate and form a solid mesh. The tissue starts to dehydrate and vessels begin to shrink at temperatures above 70°C.^1^ The combination of coagulation and shrinkage leads to vessel occlusion.

However, it often happens that the tips of the forceps stick to the vessel, and the vessel is torn open again. This can lead to several repetitions of forceps application until the bleeding finally stops. In addition, the tissue surrounding the vessel is also heated, which can result in severe neurological damage when functional brain tissue or nerves are affected. For operations in the vicinity of high-risk structures, intraoperative neurophysiological monitoring is performed to ensure that no neurological impairments occur during coagulation. The high alternating current of the bipolar forceps disturbs the electrophysiological monitoring and therefore increases the risk of neurological damage for the patient. In addition, the high current can also interfere with brain or heart pacemakers, which poses an additional risk to patient safety.

Laser radiation for tissue ablation and coagulation, also known as photocoagulation, is already established in different surgical fields such as laparoscopy or urology. One advantage of photocoagulation is the direct heating of blood or specific tissue by the absorption of laser radiation in inter- and intracellular water or in hemoglobin (Hb) molecules. This leads to morphological changes in erythrocytes and proteins, which has been already investigated in the earlier work of Abela et al.^2^ In the first step, erythrocytes change their shape from biconcave to spherical. The membrane is partially damaged, resulting in a loss of hemoglobin. As temperature rises, the membrane dissolves completely, followed by the aggregation of small globules and the subsequent formation of a continuous solid mesh. A second effect is the dehydration of tissue and the damage of collagen structures of the blood vessel wall followed by vessel shrinkage. Gorisch and Boergen^1^ showed that heat-induced shrinkage of the vessel wall starts at temperatures above 70°C to 75°C.

Laser-based coagulation can be performed without contact, thus preventing tissue adhesion to a surgical tool as it occurs with bipolar forceps. Due to the localized heating, only half of the power is necessary for laser-based coagulation compared with the power used for HF coagulation.^3^ This also shows that the efficiency of laser-based coagulation compared with HF coagulation is higher resulting in higher precision and lower thermal damage to adjacent tissue.^4^ The laser-based process can be controlled automatically using temperature measurements^5^ or OCT guidance.^6^

There are mainly two approaches for vessel occlusion by laser-based coagulation. One approach is to use a laser source in the near-infrared spectrum, such as Nd:YAG (λ=1064  nm), thulium (λ=1940  nm) or diode laser (λ=1470  nm).^5^^,^^7^^–^^10^ These wavelengths offer a high penetration depth at the expense of low selectivity and higher thermal damage. Hutfilz et al. demonstrated an occlusion rate of 92% for the occlusion of cerebral pig vessels with a diameter of 340  μm using a thulium laser (λ=1940  nm). This occlusion rate could only be achieved using ${\mathrm{CO}}_{2}$ cooling which also reduces thermal damage and ablation.^3^

Cilip et al.^9^ investigated the combination of infrared laser coagulation and vessel compression using a transparent glass slide demonstrating the occlusion of large vessels with diameters of up to 6 mm. Later, the group presented a laparoscopic prototype using infrared laser radiation from a diode laser (λ=1470  nm) guided through the shaft of a conventional Maryland jaw sealer, which compresses the vessel. Measured temperatures at the jaws were up to 135°C, which is above damage thresholds for nerve tissue.^10^^,^^11^ Contact with the vessel at the irradiation site carries the risk of the instrument getting stuck to the vessel and tearing it open again. In addition, if the instrument absorbs a part of the laser radiation, the process becomes inefficient, and the heating of the instrument endangers the surrounding tissue.

Instead of infrared radiation, another approach is to use visible laser radiation emitted by argon laser (λ=514  nm), frequency-doubled Nd:YAG laser (λ=532  nm), or laser diodes (λ=450  nm). These wavelengths are strongly absorbed by hemoglobin, which leads to a high selectivity and a lower thermal damage zone. Therefore, a lower power is sufficient for coagulation^6^ using visible laser radiation compared with infrared laser radiation. A disadvantage is the low penetration depth of about 50  μm or less.^12^ The impact of a low penetration depth on vessel occlusion has not yet been systematically investigated. Chang et al. demonstrated in mice that 450 nm laser radiation can be used to efficiently coagulate blood leakage from a small punctured hole in the mouse ear.^6^ Beyond this work, there are no investigations on blood vessel occlusion with 450 nm continuous-wave laser diodes. In particular, the investigation of vessel occlusion in dependence on vessel diameter and irradiation duration should show the role of vessel shrinkage and heat conduction in efficient vessel occlusion.

## 2 Materials and Methods

### 2.1 Experimental Setup

**Fig. 1:**
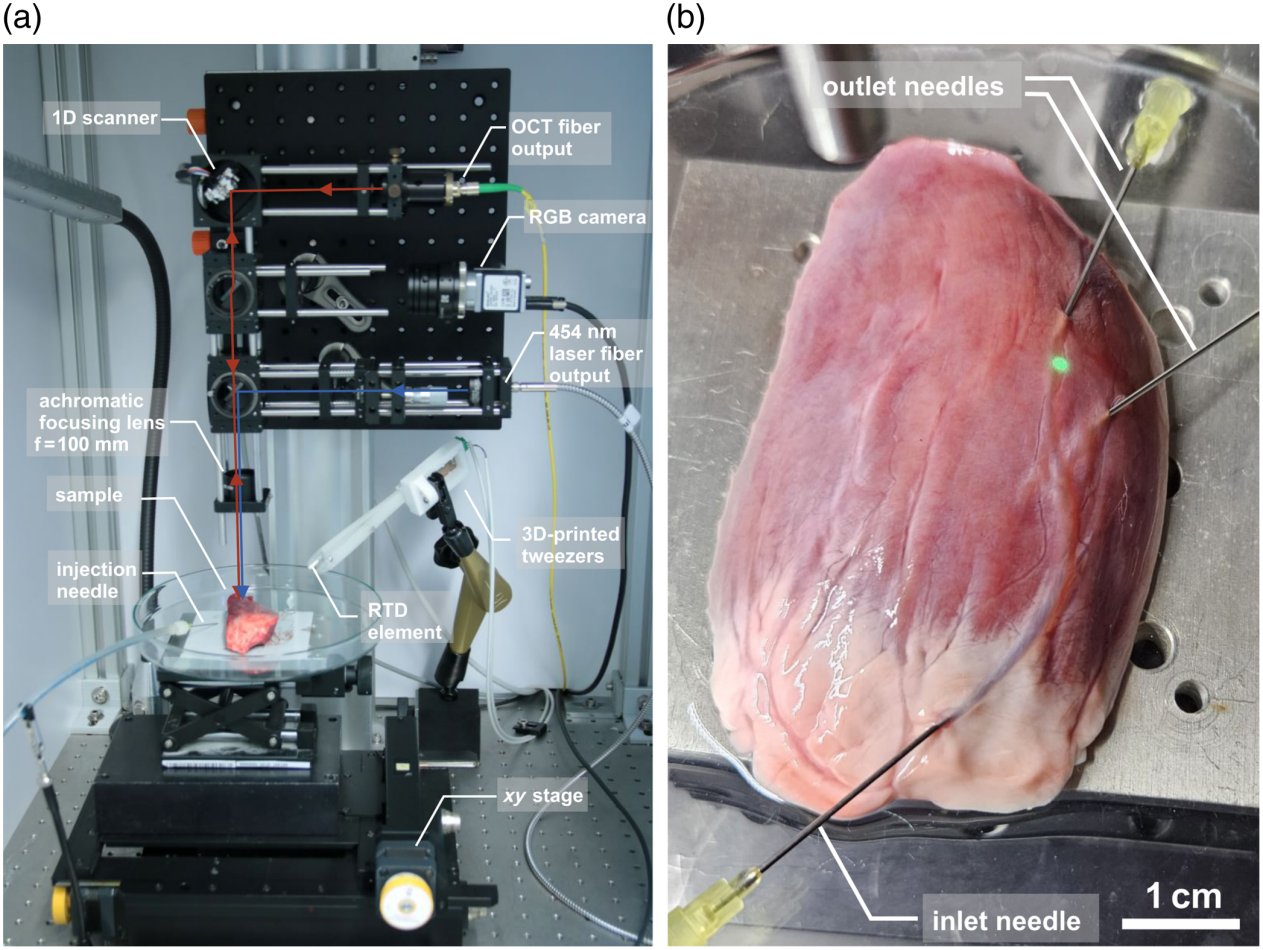
(a) Experimental chamber for laser-induced occlusion of blood vessels, including the optical setup with coaxially aligned OCT beam, RGB camera and coagulation laser, fluidic system for controlled blood flow injection, and tweezers with RTD elements for temperature measurement. (b) Part of a pig’s heart as a vascular model with injected needles for rinsing with NaCl solution and blood supply.

To investigate the laser coagulation process on blood vessels, we used a continuous-wave laser source, model FC-450-15W-FC400, from Frankfurt laser company with a wavelength of 454 nm and an available power of up to 15 W. The laser beam is fiber coupled into a fiber with 400  μm core diameter and guided to the optical setup for coagulation experiments. In Fig. 1 the experimental chamber, optical setup, computer-controlled xy-stage, petri dish for the sample, fluidic system, and 3D-printed tweezers with resistance temperature detector (RTD) elements are shown.

The optical setup combines the coagulation laser with a measurement beam for cross-sectional measurements of the blood vessel by optical coherence tomography (OCT). The OCT system is a frequency domain OCT with a central wavelength of 835 nm and a measurement rate of up to 50 kHz developed by Fraunhofer ILT. The OCT beam is deflected by a 1D planar galvanometric scanner to measure the cross-section of the blood vessel during coagulation. An analog control module from MediaLas controls the scanning mirror. For fine positioning of the laser focus on the blood vessel, we use a computer-controlled xy-stage and an RGB camera that is coaxially aligned to the blue laser. The blue laser is collimated behind the fiber output and the beam diameter is adjusted by a telescope. The OCT and coagulation beam are focused by an achromatic focusing doublet lens with a focal length of 100 mm. The OCT beam has a spot size on the sample of 30  μm. The focus diameter of the coagulation laser used in the experiments varies between 1 and 1.9 mm. The intensity profile of the beam is nearly top-hat (multimode) to get a homogeneous irradiation.

An infusion bottle with blood solution is attached to a tower that is adjustable in height to set the blood pressure. The height of the bottle is adjustable between 50 and 160 cm above the sample to mimic physiological blood pressure. The blood pressure in arterioles and small arteries is about 45 mmHg which corresponds to ∼60  cm height.^13^ At maximum height, a pressure of 120 mmHg is obtained, which corresponds to the systolic blood pressure in large arteries. Blood flow is measured with a flow sensor, model SLF3S-1300F, from the company Sensirion, Stäfa, Switzerland, which can measure a flow rate of up to $40\;\mathrm{ml}\;\min^{-1}$. An injection needle with a diameter of 800  μm is used to connect the vessels to the blood flow.

In addition, a prototype of tweezers is developed and 3D-printed with selective laser sintering (SLS) from the company name is Materialise, Löwen, Belgium. Two RTD elements of model no. 1PT100KN1515 from the company Omega Engineering, Deckenpfronn, Germany with two leads are integrated into the tweezer tips. With the temperature sensors, the heating of the surrounding tissue can be measured.

### 2.2 Vascular Model

A fresh pig heart from the local butchery is used as a vascular model for the coagulation experiments. The superficial coronary vessels are mainly arterioles and arteries with diameters between 50  μm and 2 mm that are easily accessible. Individual vascular trees were prepared from the heart and connected to the fluidic system for easier handling. The experiments are carried out on the first and second day after slaughter to avoid major changes in the sample due to biological decomposition processes. Figure 1(b) shows an example of a sample connected to the fluidic system.

For the coagulation experiments, two different solutions were used. The first solution is prepared from hemoglobin powder obtained from bovine blood. The hemoglobin powder can be dissolved in a NaCl solution at a concentration of $20\;gl^{-1}$. This corresponds to about 13% of the natural hemoglobin concentration in adult human blood. Therefore, a correspondingly lower absorption is expected. Because of the lower absorption, the process dynamics can be investigated using OCT measurements. The hemoglobin solution is also easily accessible and has a longer shelf life compared with fresh blood.

The second solution used is fresh pig’s blood from a local slaughterhouse. Artery and venous blood were taken. Sodium citrate was added to the blood to inhibit coagulation. Citrate binds to calcium ions and interrupts the natural coagulation cascade. The experiments were carried out in the first two days after slaughter to avoid major changes in the sample due to biological decomposition processes.

### 2.3 Experimental Procedure

First, the laser parameters required for reliable vascular occlusion are estimated in experiments with drops of citrated whole blood. After the analysis of coagulation in the blood drops, the vessel occlusion is examined using the superficial arteries of pig hearts.

#### 2.3.1 Coagulation of blood

Individual blood droplets of citrated blood were scanned with the OCT beam to determine their height and shape. The blood is irradiated for a short duration of t<1  s with different intensities to identify a suitable value for fast coagulation without damaging the blot clot. There is an intensity threshold $I_{\mathrm{coag}}$, where coagulation occurs and a threshold for ablation $I_{\mathrm{abl}}$. Using a different value of I with $I_{\mathrm{coag}}<I<I_{\mathrm{abl}}$ for coagulation, the size of the blood clot is investigated in dependence on the irradiation duration t with 0.5  s<t<5  s. The time-dependent formation of the blood clot is observed with the OCT system. After irradiation, the blood clot is separated from the remaining liquid blood, and its thickness is measured with OCT. A refractive index of n=1.37 is assumed for measurements, which is taken from the literature.^14^

#### 2.3.2 Blood vessel occlusion

To observe the vessel shrinkage by OCT, experiments were first carried out with the hemoglobin solution. As this solution has only 13% of the natural hemoglobin concentration, the absorbance A is only 13% of the absorbance in whole blood with $A=\ln(I_{0}/I)$. The intensity in these experiments was $I=18.8\;W\;{\mathrm{cm}}^{-2}$, and the irradiation time was t=5  s. The results of the experiments on droplets of citrated whole blood and the hemoglobin solution were used to identify suitable laser parameters for vascular occlusion with whole blood.

For the systematic investigation of vessel occlusion with whole blood, an intensity of $I=32.1\;W\;{\mathrm{cm}}^{-2}$ was examined for two irradiation durations: t=1  s and t=3  s. Table 1 shows the laser parameters for these experiments.

**Table 1:** Experimental parameters for the coagulation experiments with two different irradiation durations.

Parameter		Setting I	Setting II	Setting III	Setting IV
focal length	f (mm)	100	100	100	100
focus diameter	d (mm)	1.9	1.9	1.9	1.9
laser power	P (W)	0.91	0.91	0.91	0.91
intensity	I ($W\;{\mathrm{cm}}^{-2}$)	32.1	32.1	32.1	32.1
irradiation duration	t (s)	1	1	3	3
OCT power	$P_{\mathrm{OCT}}$ (mW)	0.4	0.4	0.4	0.4
OCT measurement frequency	$f_{\mathrm{OCT}}$ (kHz)	5	5	5	5
blood pressure	p (mmHg)	45	120	45	120
flow rate	Q ($\mathrm{ml}\;\min^{-1}$)	1…5	5…10	1…5	5…10

The experiments were carried out with a blood pressure of 45 and 120 mmHg. In total, 99 vessels were investigated for laser-based blood vessel occlusion. At first, a vascular tree was dissected from the heart and rinsed with NaCl solution to ensure that the vessel’s lumen was free of particles. The vessel is scanned with the OCT beam, adjusting the scanning amplitude, scanning velocity, and measurement frequency of the OCT to the vessel size and signal intensity. A needle connects the largest vessels of the vascular tree to the infusion bottle with blood, and the examined vascular tree is filled with blood. The flow rate in front of the vessel tree is measured with the flow sensor. It depends on the individual vessel cord, in particular the vessel sizes and number of branches. Therefore, the flow rates differ in the range of 1 and $10\;\mathrm{ml}\;\min^{-1}$. The vessels on the sample were selected in randomized order regardless of their size. After irradiation, the vessel is evaluated with respect to successful occlusion. The evaluation is done using flow measurement and the absence of blood leakage from the vessel. Visible carbonization on the tissue is also noted.

The height of the vessel h and its width w can be determined from the cross-sections measured by OCT considering the scanning parameter and the refractive index of 0.9% NaCl solution, where n=1.33.^15^ The vessel diameter assumed is the equivalent diameter d, calculated from the height h and width w as follows:

$$d=\sqrt{w\cdot h}.$$ (1)

The occlusion rate for an individual set of parameters is defined as the number of successful occlusions $N_{i,\text{sealed}}$ divided by the total number of vessels in this parameter group $N_{i}$

$$O_{R,i}=\frac{N_{i,\text{sealed}}}{N_{i}}.$$ (2)

Due to the low sample size in each parameter group, the distribution is assumed as binomial distribution with a standard deviation $\Delta O_{R,i}^{2}$ and an error $\Delta O_{R,i}$ of

$$\Delta O_{R,i}^{2}=N_{i}\cdot O_{R,i}(1-O_{R,i}),$$ (3)

$$\Delta O_{R,i}=\sqrt{N_{i}\cdot O_{R,i}(1-O_{R,i})}.$$ (4)

## 3 Results and Discussion

### 3.1 Coagulation of Blood

Blood is successfully coagulated at an intensity of $I=15.7\;W\;{\mathrm{cm}}^{-2}$ and irradiation times between 0.5 and 4 s. A longer irradiation time led to a larger blood clot (see Table 2). At an intensity of $I=21.1\;W\;{\mathrm{cm}}^{-2}$, the blood clot bursts even at short irradiation times of 0.5 s. One assumption is that the high intensity causes rapid coagulation and the remaining water in the coagulum evaporates. The high pressures during vaporization can destroy the coagulum. Similar effects are also used in soft tissue ablation.

**Table 2:** Laser parameters and results of the experiments of blood coagulation.

Parameter		E1	E2	E3	E4	E5
intensity	I ($W\;{\mathrm{cm}}^{-2}$)	15.7	15.7	15.7	15.7	15.7
irradiation durations	t (s)	0.5	1	2	3	4
blood droplet thickness	$d_{f}$ (μm)	361	889	770	806	765
blood coagulum thickness	$d_{c}$ (μm)	260	286	235	390	488

**Fig. 2:**
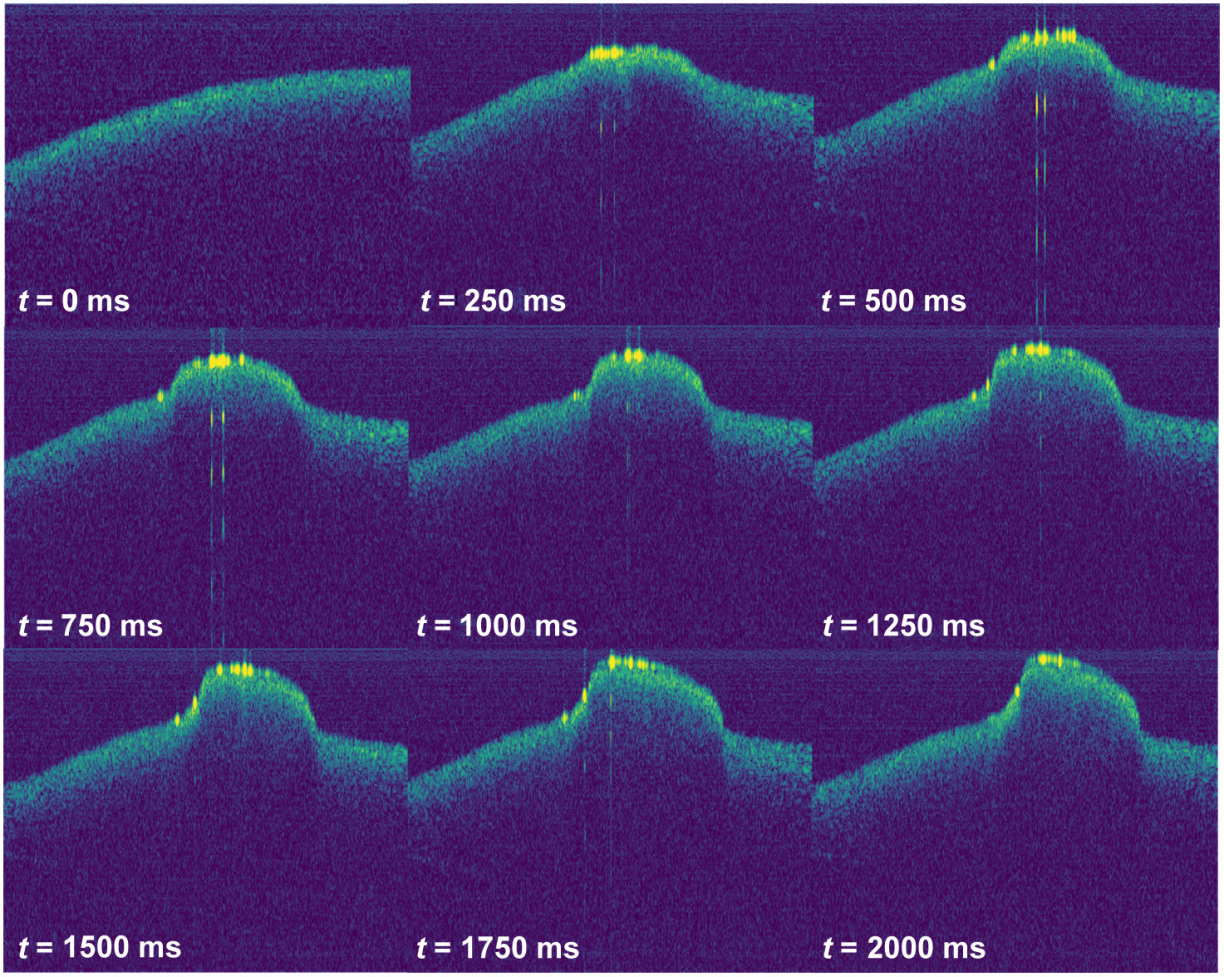
Time series of the coagulation of a blood droplet at 454 nm wavelength and an intensity of $15.7\;W\;{\mathrm{cm}}^{-2}$ using OCT measurements.

A time sequence of OCT images from the coagulation process on blood droplets using an intensity of $15.7\;W\;{\mathrm{cm}}^{-2}$ is shown in Fig. 2. Coagulation starts within the first 250  ms, which is indicated by the higher reflectance of the OCT beam at the surface of the blood droplets. The surface becomes rough and the transmission through the blood decreases. At some locations on the coagulum, the high reflectance leads to artifacts in the OCT image visible as multiple reflectances underneath the surface. The largest growth of the blood clot is observed at the beginning of the process. Then it slowly increases, until after 1.75 s, only a very small change in size is visible. Even after 4 s of irradiation, it was not possible to coagulate the whole height of the blood droplet which was 800  μm. The maximum height of the blood clot was 488  μm in 4 s, which is much higher than the penetration depth in blood of 50  μm for a wavelength of 454 nm. This leads to the assumption that heat conduction in the blood plays a crucial role in the formation of large blood clots.

In summary, longer irradiation times lead to a larger blood coagulum, which increases the probability of occlusion of large vessels up to 1 mm in diameter. The effect of blood coagulation alone will not lead to reliable vessel occlusion within short irradiation durations. Thermal shrinkage of the vessel is required, as described in Sec. 1.

### 3.2 Blood Vessel Occlusion

**Fig. 3:**
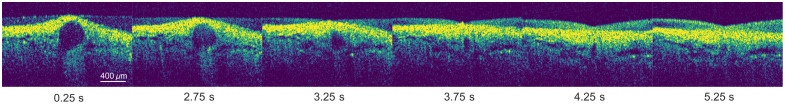
Time series of OCT images of coronary artery occlusion flushed by hemoglobin solution during laser irradiation with an intensity of $18.8\;W\;{\mathrm{cm}}^{-2}$ and 5 s irradiation duration. The first signs of vessel shrinkage occurred at 2 s. The complete forming of a coagulum takes more than 5 s.

Using the hemoglobin solution, blood vessel occlusion could only be achieved for small vessel diameters of 200  μm or less. The hemostasis of larger blood vessels was not even possible for longer irradiation durations of up to 5 s. Because of the lower scattering in Hb solution, it was possible to observe the vessel shrinkage during irradiation. Figure 3 shows a time sequence for occlusion of a blood vessel with a diameter of 380  μm, an intensity of $18.8\;W\;{\mathrm{cm}}^{-2}$ and an irradiation duration of 5 s. The images show a rapid reduction in vessel volume within the first 3.75 s. The velocity of shrinkage decreases with increasing coagulation duration. After 5.25 s no blood vessel volume is visible in the OCT image.

The slow vessel shrinkage and the difficulty in the coagulation of large vessels can be a consequence of the lower absorption. The absence of red blood cells can probably also lead to a smaller coagulum that only consists of coagulated proteins. In recent years, the important role of red blood cells in hemostasis has been demonstrated by several researchers. In addition to their properties of adhering to the vessel wall, they contribute to the enlargement and solidification of the coagulum.^16^

**Fig. 4:**
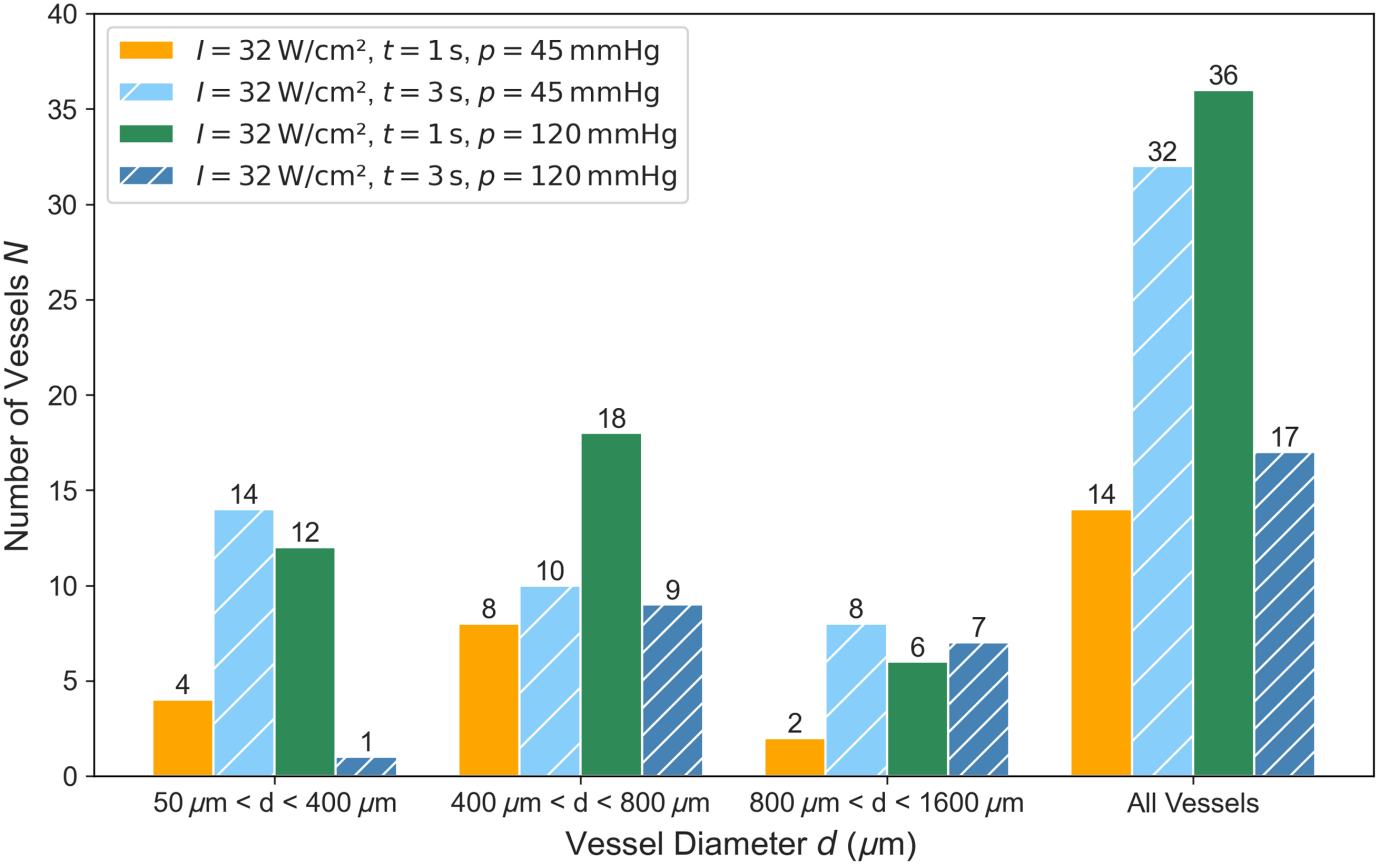
Number of vessels divided into three groups of vessel diameters with four different experimental parameter settings.

**Fig. 5:**
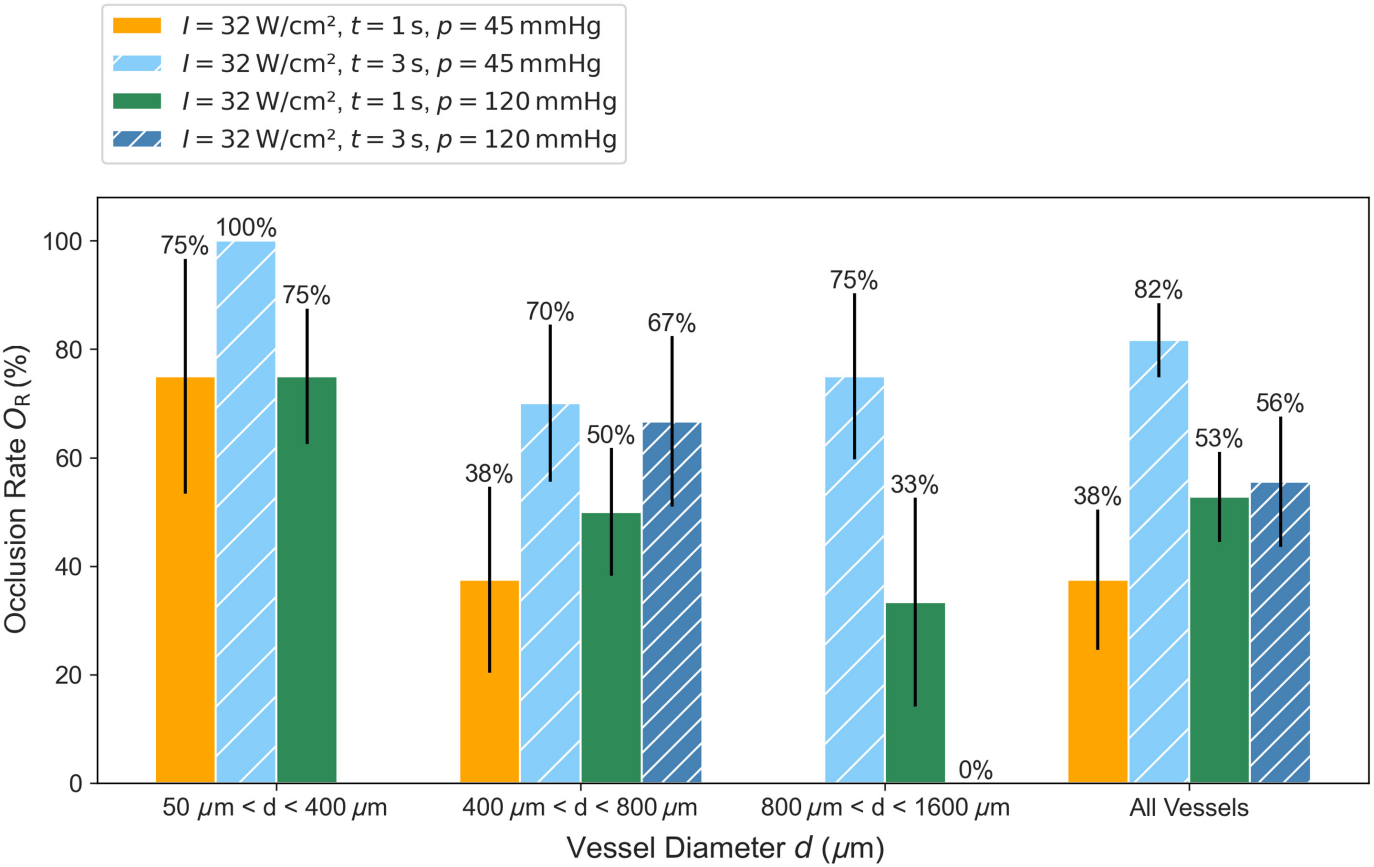
Occlusion rates in dependence on vessel diameter, irradiation time, and pressure at the needle output of the blood infusion system.

The 99 vessels that were part of the experiments can be divided into three groups of vessel diameter: One group with small vessel diameters ranging from 50 to 400  μm, a second group with vessel diameters ranging from 400 to 800  μm, and a third group ranging from 800 to 1600  μm. These groups can be further divided by the four-parameter settings of the experiments with different irradiation durations and blood pressures. The number of vessels in each of the 12 groups is shown in Fig. 4. For reasons of statistical significance, only groups containing four or more results are included in the analysis of the occlusion rate. The occlusion rates $O_{R}$ obtained in dependence of the vessel diameter d, the irradiation duration t, and the blood pressure p are shown in Fig. 5.

The highest occlusion rate achieved was 100% for small vessels with an intensity of $32.1\;W\;{\mathrm{cm}}^{-2}$, an irradiation time of 3 s and a blood pressure of 45 mmHg. Due to the small number of vessels, no significant difference in the occlusion rate could be found between an irradiation duration of 1 and 3 s. The occlusion rate for small vessels with a high pressure of 120 mmHg is also comparable to that of 45 mmHg, indicating that the coagulum can also be formed under higher blood pressure conditions.

For midsize vessels between 400 and 800  μm in diameter, there is a higher occlusion rate with an irradiation duration of 3 s than for 1 s, which agrees well with the results of Sec. 3.1. A longer irradiation duration leads to a larger coagulum, which in combination with vessel shrinkage leads to vessel occlusion for vessels up to 800  μm diameter. Between low and high blood pressure, no significant difference could be determined. For larger vessels, there is a clear difference between the occlusion for a pressure of 45 mmHg and one of 120 mmHg. These findings also agree with the work of Katta et al.^17^ investigating the effect of flow rate and shear stress on occlusion of larger vessels. The flow rate and the resulting shear stress seem to play an important role in the reliable occlusion of larger vessels. In addition, the higher pressure of 120 mmHg leads to a higher tension visible in vessel dilatation that counteracts the vascular shrinkage. Furthermore, the greater expansion of the vessel due to the higher pressure also leads to a larger absorption volume. The combination of a long irradiation duration of 3 s and a high pressure of 120 mmHg leads to the highest carbonization rate of 23%. The carbonization can also damage the coagulum. This could be an explanation for the difference in the occlusion rate for 1 and 3 s irradiation duration using the higher pressure.

In total, a decrease in the occlusion rate can be found with larger vessel diameters in all four groups. Using an irradiation duration of 3 s for a pressure of 45 mmHg, an overall occlusion rate of 82% could be achieved.

#### 3.2.1 Thermal damage zone

In some of the performed experiments, the temperature has been measured with RTDs integrated into the tips of tweezers at a distance of ∼2  mm from the vessel. At this distance, only a small temperature rise occurred, with a maximum temperature of 28°C. The room temperature and the sample temperature were 23°C. Considering a body temperature of 37°C, this temperature rise would lead to a temperature of 42°C. Although cell necrosis begins at temperatures above 43°C, it occurs only for long exposure times that exceed 30 min.^18^ Current studies identify temperature thresholds between 50°C and 60°C for damaging cranial nerves if this temperature is applied for a few seconds.^11^ Therefore, the temperature rise that is induced by the laser process falls below the critical values for nerve damage by a factor of 2.

**Fig. 6:**
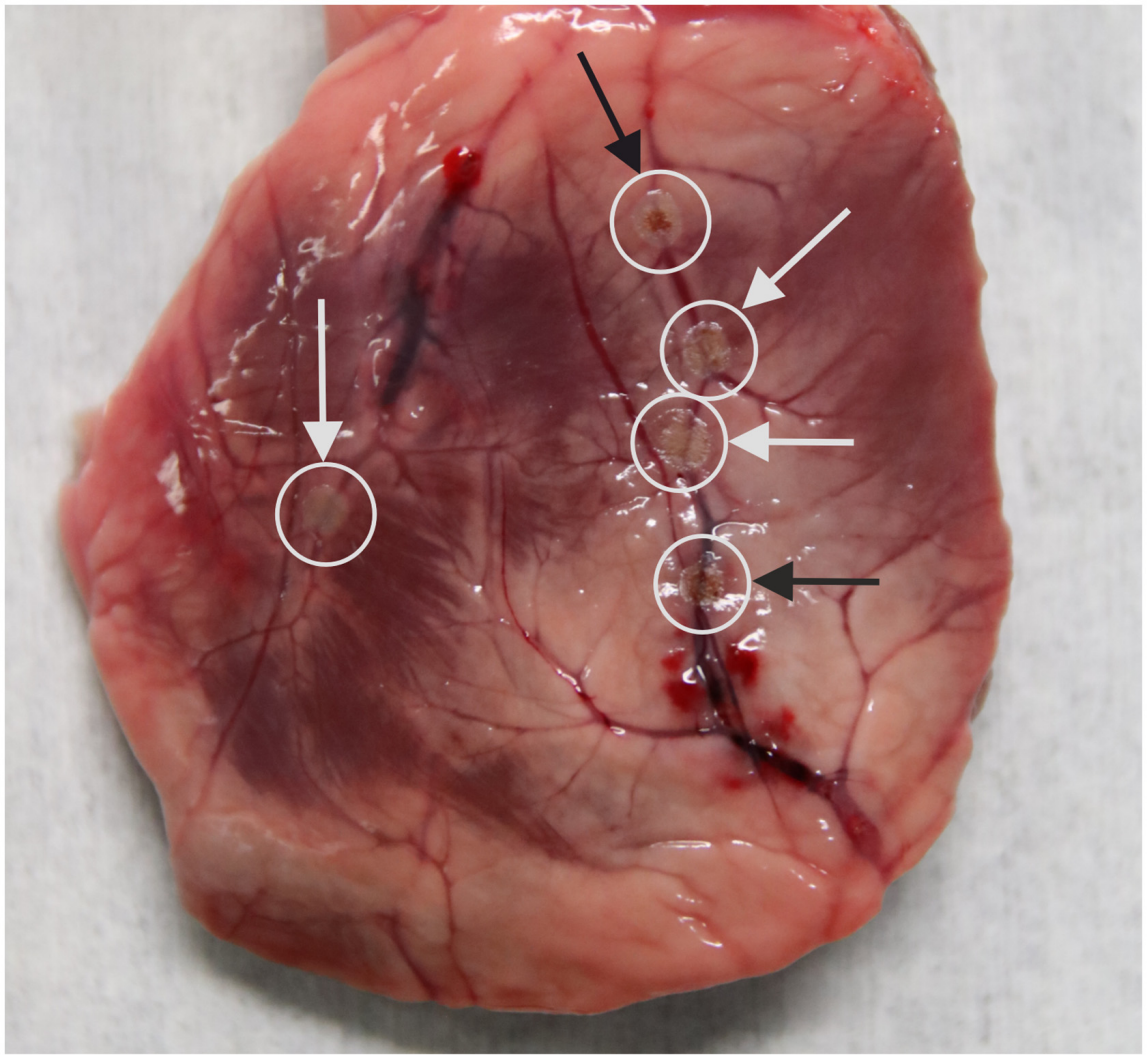
Superficial arteries of a pig’s heart coagulated with laser radiation of 454 nm wavelength for an irradiation duration of 3 s. Vessel shrinkage is visible in all coagulation locations. Carbonization occurs in some cases and is marked with a black arrow.

The temperature measurements agree well with the observations of thermal damage in the coagulation zone. In Fig. 6, a sample with five coagulation zones is shown. The coagulation zones are marked with white and black arrows, whereby white arrows indicate coagulation zones without visible carbonization and black arrows indicate carbonized areas. Even in the cases of visible thermal damage, the damage is limited to a small area of 1 mm in diameter in the center of the vessel, whereas the surrounding tissue is coagulated but not carbonized. To measure the thermal damage zones more precisely, histological examinations would be necessary.

## 4 Conclusion and Outlook

The experimental investigations show that photocoagulation of large vessels with a diameter of up to 1.5 mm using a continuous-wave laser diode of 454 nm wavelength is possible. Occlusion rates of 82% could be achieved with an intensity of $32.1\;W\;{\mathrm{cm}}^{-2}$, an irradiation duration of 3 s, and a pressure at the output of the injection needle of 45 mmHg. As the blood pressure of only 45 mmHg is mostly common in arteries smaller than 500  μm and veins of about 1 mm, future research aims to increase occlusion rates, whereas higher blood pressure of at least 120 mmHg is applied.^19^ The experiments show that longer irradiation times lead to a larger blood coagulum and a higher occlusion rate of midsize and large vessels by thermal conduction. Carbonization occurred in 18% of the irradiated vessels, whereby only the inner area of the irradiation area with about 1 mm diameter is affected. At a distance of ∼2  mm from the vessel, the temperature rise stays below the critical thresholds for thermal nerve damage.

To further improve the occlusion rates of large vessels, under a pressure of 120 mmHg, longer irradiation times should be considered. Another alternative would be to choose a wavelength with a higher absorption length in the hemoglobin to achieve a more uniform energy deposition over the entire thickness of the vessel. As strong bleeding can lead to a thick blood film on top of the ruptured vessel, continuous rinsing with NaCl solution would be required for wavelengths with high hemoglobin absorption. To compare the strength of the vessel seals from our approach with those done by conventional instruments, burst pressure measurements should be integrated into future studies. Overall, this work demonstrates a laser process for photocoagulation that can improve patient safety during neurosurgical procedures as the thermal damage zone is small, and efficient occlusion of the vessels is possible without interfering with electrophysiological monitoring.

## Data Availability

Data generated and analyzed during this study are not publicly available due to confidentiality agreements with research collaborators but are available from the corresponding author on reasonable request.
